# Decision trees: from efficient prediction to responsible AI

**DOI:** 10.3389/frai.2023.1124553

**Published:** 2023-07-26

**Authors:** Hendrik Blockeel, Laurens Devos, Benoît Frénay, Géraldin Nanfack, Siegfried Nijssen

**Affiliations:** ^1^Department of Computer Science, KU Leuven, Leuven, Belgium; ^2^Institute for Artificial Intelligence (Leuven.AI), KU Leuven, Leuven, Belgium; ^3^Faculty of Computer Science, Université de Namur, Namur, Belgium; ^4^ICTEAM, UCLouvain, Ottignies-Louvain-la-Neuve, Belgium

**Keywords:** decision trees, ensembles, responsible AI, machine learning, learning under constraints, explainable AI, combinatorial optimization

## Abstract

This article provides a birds-eye view on the role of decision trees in machine learning and data science over roughly four decades. It sketches the evolution of decision tree research over the years, describes the broader context in which the research is situated, and summarizes strengths and weaknesses of decision trees in this context. The main goal of the article is to clarify the broad relevance to machine learning and artificial intelligence, both practical and theoretical, that decision trees still have today.

## 1. Introduction

Decision trees, and ensembles of them (forests), are among the best studied and most widely used tools for machine learning and data science. In their basic form, they are covered in just about every introductory course on these fields, and the extensive literature on them is covered by many surveys. However, there is plenty of application potential for decision trees beyond the commonly known uses. The existing surveys typically do not zoom in on this, or when they do, they zoom in on one particular type of use.

The purpose of this review is to complement the literature by taking a step back and providing a higher-level overview of decision tree technology and applicability, covering the broad variation in it. The review focuses on answering questions such as: what types of decision trees exist (beyond the well-known classification and regression trees), what can they be used for, what roles can decision trees play in an age that is dominated by deep learning? It describes the landscape and evolution of decision tree research in a way that is roughly chronological, starting with the earlier research, which focused mostly on decision trees as predictive models, and gradually moving toward more recent work on learning or exploiting decision trees in the context of what is currently often referred to as responsible AI: the study of AI systems that are fair, transparent, and safe.

The review does not aim at surveying the research field in the traditional sense. For some topics, it points the reader to existing surveys; at other times, concrete publications are referred to as illustrative examples of the topics being discussed. Nevertheless, in a few cases, where we believe a subject is insufficiently covered by existing surveys, a more detailed overview of work is given.

The review starts (Section 2) with an overview of the basics of decision trees: classification and regression trees as they were originally envisioned, methods for learning them, variants and ensembles. What connects all this work is that decision trees are seen as predictive (and sometimes explanatory) models. However, decision trees can be used beyond the classification and regression context: they can be used for multi-label learning, multi-instance learning, (predictive) clustering, probability and density estimation, and other purposes, both standalone and integrated in other methods. Section 3 covers such uses.

Section 4 briefly discusses different algorithmic approaches to learning decision trees: besides the standard heuristic approaches, incremental and distributed variants have been proposed, as well as approaches based on non-greedy optimization and continuous parameter optimization. This section includes an extensive discussion of exhaustive methods that search for optimal decision trees (given some optimization criterion)—an NP-hard problem that due to recent advances in solver technology has received much interest. This discussion focuses on providing insight in the different approaches and how they relate to each other.

Section 5 discusses how background knowledge in the form of formal constraints can be incorporated in decision trees, either by imposing the constraints on the model at learning time, or by verifying given models. Learning models under constraints is currently receiving increasing interest, partly because constraints are useful to enforce other properties than accuracy and interpretability, such as robustness and fairness, and partly because the technology now allows it: the current state of the art in greedy and exhaustive search methods facilitates the creation of methods that take constraints into account.

In Section 6, we provide an overview of how decision tree based methods play a role in the current research on Responsible AI, with a specific focus on robustness, fairness, and explainability. This section covers mostly recent work.

Section 7 offers a brief look forward, mentioning challenges and perspectives, and Section 8 concludes.

## 2. Decision trees and forests: the basics

This section discusses the basics of decision trees. It focuses mostly on the area as it was seen by the end of the 20th century, and is meant to set the background for the later sections. We introduce the concept of decision trees, the greedy learning methods that are most commonly used for learning them, variants of trees and algorithms, and methods for learning ensembles of trees. The section ends with an overview of strengths and weaknesses of decision trees and forests.

### 2.1. Decision trees

**Figure 1:**
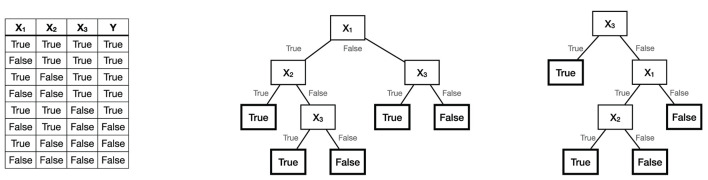
The Boolean function *Y* = *X*_1_ ∧ *X*_2_ ∨ *X*_3_, and two decision trees representing it.

A decision tree represents a procedure for computing the outcome of a function *f*(*x*). The procedure consist of repeatedly performing tests on the input *x*, where the outcome of each test determines the next test, until *f*(*x*) is known with certainty. Figure 1 shows a function in tabular format and two different decision trees that represent it.

Decision tree learning refers to the task of constructing from a set of (*x, f*(*x*)) pairs, a decision tree that represents *f* or a close approximation of it. When the domain of *x* is finite, the set of pairs can in principle be exhaustive, but more often, the set is a sample from a (possibly infinite) domain X. In that case, rather than finding a tree that approximates *f* on the data set, one may try to find a tree that approximates *f* over the whole domain.

In a slightly generalized setting, the set of pairs may be of the form (*x, y*) where *y* is determined only probabilistically by *x*; for instance, *y* may depend also on unobserved variables, *y* = *f*′(*x, u*). The task is then to learn a tree that represents a function *f*(*x*) that closely approximates *f*′(*x, u*) for any choice of *u* (on average, in the worst case, or using some other aggregation criterion).

Apart from finding a good approximation, additional criteria may exist. For instance, the task may be to find the simplest decision tree that represents the function. It is known that finding the smallest decision tree (in terms of number of nodes) that perfectly fits a given dataset is NP-hard (Hyafil and Rivest, 1976).

The output of a tree for a given *x* is often called its prediction for *x*. Decision trees that predict nominal or numerical variables are respectively called **classification trees** and **regression trees**. An important property of decision trees, in the context of machine learning, is that the prediction is the result of a simple and easy-to-interpret computation (a relatively short series of tests). Because of this, trees are said to be *interpretable*.

### 2.2. Recursive partitioning

Decision trees became prominent in machine learning and data analysis around the 1980s, when popular decision tree learners were developed more or less in parallel in the computer science community (e.g., ID3 Quinlan, 1986 and its many subsequent improvements) and in the statistics community (CART Breiman et al., 1984). While differing in details, these learners all make use of the same basic procedure, namely **recursive partitioning** (also known as “top-down induction of decision trees”).

Recursive partitioning works as follows. Given a dataset *D* containing pairs (*x, y*), a test that can be performed on individual instances *x* is chosen, the dataset is partitioned according to the outcome of this test, and this procedure is repeated for each subset thus created. This continues until no further partitioning is needed or possible. This procedure relies on two important heuristic criteria: how to choose the test, and when to stop.

The chosen test is typically selected from a set of candidate tests, and is that test that is deemed “most informative” with respect to the value of *y*. A test is maximally informative when, given the outcome of the test, the *y* value is known exactly. For numerical *y*, the average variance of *y* within each subset of the partition is often used as an indicator for the informativeness of the test. For nominal *y*, measures based on information theory are sometimes used, such as information gain (the difference between the entropy of *y* and its average conditional entropy given the test outcome).

Using the “most informative” test is motivated by a preference for finding short decision trees (which require few tests to come to a decision), but there is no guarantee that any of the above measures indeed lead to the shortest possible tree. From that point of view, the procedure is heuristic. Early research on decision trees has explored many different variants of the heuristics, without unearthing a universally preferable one: see, e.g., Murthy (1998, Section 3.1.1).

It is clear that a subset need not be partitioned further when only one value for *y* remains, but it may be better to stop splitting even earlier, as for small sets, further splitting may cause overfitting.^1^ Therefore, learning algorithms usually stop splitting when a set is too small to carry any statistical significance. Alternatively, an algorithm may keep splitting but prune the tree afterwards. Again, many variants of stopping criteria and pruning procedures have been explored, without any one consistently outperforming the rest, though on individual datasets there may be substantial differences in performance (Murthy, 1998).

For a more extensive discussion of the many variants of decision tree learners that had been proposed by the end of the 20th century, we refer to the comprehensive survey by Murthy (1998). A recent survey by Costa and Pedreira (2023) focuses on progress after 2010. Observing that choosing the right variant can sometimes make a difference, Barros et al. (2015) propose a method for constructing tailor-made recursive partitioning algorithms that chooses components optimally for a given dataset.

Recursive partitioning is probably the best known and most often used method for learning decision trees, but it is not the only one: Section 4 of this text discusses alternatives. Especially in recent years, these alternative methods are gaining interest.

### 2.3. Variants of classification and regression trees

Decision trees are often used with tabular data, where each instance is described using the same set of input variables. Tests are often univariate (based on a single variable), and in the case of numerical inputs, based on a dichotomy (value above or below some threshold). However, it is perfectly possible for learners to consider multivariate tests. An example of this are so-called **oblique decision trees**, which use a threshold on a linear combination of input variables; this results in straight-line boundaries between the subsets that are not necessarily axis-parallel (Murthy et al., 1994).

In the above, we assumed that decision trees make the same prediction for all instances in a given leaf. However, variants exist that store in a leaf a function, rather than a single value, for the prediction. **Model trees**, for instance, store a linear model in a leaf, rather than a constant, so that the model represented by the tree is piecewise linear, rather than piecewise constant. M5 (Quinlan, 1992) is a well-known example of such a system.

Trees can also be used with non-tabular data, such as graphs, relational databases, or knowledge bases. The only requirement is that tests can be defined on individual instances. This has led to the development of **relational** (a.k.a. *structural* or *first-order-logic*) decision trees (Kramer, 1996; Blockeel and De Raedt, 1998).

All the above variants of decision tree learning still fit under the header of classification and regression trees. Section 3 will focus on decision trees that serve other purposes, such as clustering or density estimation.

### 2.4. Ensembles of trees: decision forests

**Ensemble methods** reduce the effect of random artifacts in the training set or learning procedure by repeating the learning process multiple times, and creating a meta-model that makes predictions by aggregating the predictions of the individual learned models. To construct multiple trees from a single data set, one can use bootstrap aggregating, a.k.a. **bagging**: each individual tree is trained on a random sample of |*T*| instances drawn with replacement from the training set *T*, and the prediction of the ensemble is the average (for regression) or mode (for classification) of the individual predictions. Ensembles of decision trees constructed in this way significantly outperform single decision trees in terms of accuracy (Breiman, 1996). The **Random Forests** method (Breiman, 2001) is a variant of this in which the test put in each node is the best from a randomly chosen subset (rather than all) of the possible tests. The additional randomness thus introduced typically increases the performance of the ensemble as a whole. More recently, **gradient boosting** (Friedman, 2001) have become increasingly popular: here, additional trees are added to the ensemble in a way that mimics gradient descent in the prediction space. At the time of writing, an implementation of gradient boosting called **XGBoost** (Chen and Guestrin, 2016) is widely considered^2^ to be the method of choice when learning from tabular data: it is fast, easy to use, and very often outperforms other methods in terms of predictive accuracy. Grinsztajn et al. (2022) confirm this by means of thorough experimental verification.

The above are probably the best known types of ensemble methods, but many other exist. **Stacking** (e.g., Ženko et al., 2001) is a variant that learns to combine the votes of individual learners, rather than using a fixed voting mechanism to combine them; to this aim, a separate learner is stacked on top of the others. **Alternating decision trees** (ADT) (Freund and Mason, 1999) are trees that, besides the standard test nodes, contain “prediction nodes”, which store numerical values and can have multiple test nodes as children. Instances are sorted simultaneously to all children of a prediction node, and the prediction is the sum of the prediction nodes on all paths it follows. Thus, an ADT essentially combines the predictions of a set of decision trees, and can be seen as a compact representation of an ensemble.

Noteworthy overviews on tree ensembles include Zhou (2012) (an insightful and at the time of writing quite comprehensive view of the field); Criminisi and Shotton (2013) (which discusses a broad variety of uses of decision forests in the context of image processing); and recent surveys by Sagi and Rokach (2018) and Dong et al. (2020), which provide an excellent overview of ensemble methods (with a non-exclusive but substantial focus on decision tree ensembles).

### 2.5. Predictive learning with decision trees: pros and cons

Decision trees are very popular tools for predictive modeling for the following reasons. They are very easy to use: they typically require little or no tuning.^3^ They can be learned very fast: on the assumption that relatively balanced trees are learned (which most heuristics try to ensure), learning typically scales as *O*(*mn*log*n*) with *n* the number of rows and *m* the number of columns in the data table (i.e., only a factor *O*(log*n*) worse than scanning the table once). Under the same assumption, prediction requires *O*(log*n*) tests, which typically means a few dozen CPU instructions, per instance. Ensembles typically multiply this by a single order of magnitude, or less: e.g., Random Forests, by selecting a subset of features for each test, substantially reduces the *m* factor, which in high-dimensional domains may compensate for the extra work of learning more trees. All this make decision trees and their ensembles extremely fast and energy-efficient, which is a major advantage when deploying models on small battery-powered devices.

Early research mostly focused on making decision trees as accurate as possible. Individual trees could never beat more complex models such as neural networks in this respect, but it is now generally acknowledged that forests can. Ensembles do give up some interpretability for this. Still, it is important to distinguish different forms of interpretability: (1) understanding the full model; (2) understanding aspects of the full model, such as which variables are important; (3) understanding a single prediction; (4) understanding the reasoning process involved in a prediction. Random Forests, for instance, are highly interpretable in the sense of 2 and 4.

Decision forests typically perform less well when learning from raw data (such as images, sound, text), where the features relevant for prediction have to be constructed and cannot be expressed as logical combinations of relatively few input features. This type of problems is what deep learning excels at.

## 3. Beyond classification and regression

Decision tree learning was originally proposed in the standard predictive learning setting, where an output variable (nominal or numerical) needs to be predicted from input variables. The algorithm is sufficiently flexible, however, to generalize decision tree learning to many other settings.

A first type of generalization is **multi-target prediction**, where a single tree predicts multiple output variables at the same time (possibly a mix of numerical and nominal variables). This setting includes prediction of set-valued variables, as in **multi-label prediction**, since sets are easily represented as binary vectors. This generalization can be achieved by simply maintaining the variance reduction heuristic from regression trees, now using variance in a higher-dimensional space. Single multi-target trees have in some contexts exhibited better performance than sets of single-target trees (Vens et al., 2008). In a further generalization, Kocev et al. (2013) studied decision tree ensembles for **structured output prediction**.

A natural extension of multi-target trees are **clustering trees**, which use variance in the input space as a heuristic, rather than variance in the output space, to learn a hierarchical clustering where each cluster is strictly characterized by a conjunction of selected attribute values. This additionally makes it possible to naturally interpolate between the predictive and clustering settings to obtain so-called **predictive clustering trees**, which form coherent clusters within which accurate prediction of the output variables is possible (Blockeel et al., 1998).

**Survival trees** are regression trees used in the context of survival analysis. The specific challenge in learning such trees is that the survival data used for training is often censored: only lower bounds are known for the labels (i.e., we do not know the exact time of death, only that up till a certain moment in time a person was still alive). Bou-Hamad et al. (2011) and Zhou and McArdle (2015) survey algorithms for learning survival trees and forests. These algorithms typically fit a hazard function to instances in a leaf of the tree, and use heuristics that either maximize heterogeneity among subsets after the split, or maximize homogeneity within them (for some homogeneity criterion that suits the context of survival analysis).

Decision trees have also been used for **ranking** and **preference learning**. As noted by Fürnkranz and Hüllermeier (2010), ranking is an umbrella term for a variety of tasks. For instance, given *n* instances and *m* classes, one may predict a preference ordering of classes for each instance (*label ranking*), or of instances for a specific class (*instance ranking*; one typically ranks the instances according to likelihood of belonging to the positive class, as in finding the most relevant webpages for a query). Training examples may be labeled with a single class (*ordinal classification*), a complete label ranking, or a partial ranking (e.g., which of two labels or instances is preferred over the other). Examples of decision tree based approaches for label ranking are Todorovski et al. (2002) and Yu et al. (2011); both learn from complete label rankings. Instance ranking with decision trees, learning from binary labels, was studied (among others) by Provost and Domingos (2003), who use probability estimation trees for the task, and Clémençon et al. (2011) who study ranking with decision trees as a task in itself.

The **multi-instance** learning setting is a binary classification setting where labels are available at the level of groups of instances rather than individual instances: a group is positive if it contains at least one positive instance, and negative otherwise. Small changes to the recursive partitioning algorithm suffice to make decision tree learning successful in multi-instance learning (Blockeel et al., 2005).

Decision trees have also been adapted for **semi-supervised** learning (Levatić et al., 2017) and learning from positive and unlabeled data (**PU-learning**) (Liang et al., 2012). In PU-learning, they have also been used for estimating the labeling rate of positive cases (Bekker and Davis, 2018), serving as an auxiliary method for any kind of PU-learners.

In the context of anomaly detection, a tree-based approach called **Isolation Forests** (Liu et al., 2008) is considered state-of-the-art for a wide range of applications. The rationale behind Isolation Forests is that with random splitting, anomalies tend to get isolated into a singleton leaf early on, so the depth of a singleton leaf is an indication of how anomalous the instance in that leaf is.

Decision trees are useful also in probabilistic settings. **Probability estimation trees** (PETs) are decision trees that predict probabilities rather than just classifying instances; that is, given *x*, they predict *P*(*y*|*x*) rather than just the *y* that maximizes it. PET learning typically benefits from less aggressive pruning than classification tree learning (Provost and Domingos, 2003; Fierens et al., 2010). **Density estimation trees** (DETs) model a density function over the input space (Ram and Gray, 2011). Both types of models have been shown to be successful at modeling conditional and joint probability densities.

PETs are particularly useful in the context of probabilistic graphical models (PGMs). They can be used to model **conditional probability functions** (instead of probability tables) (Friedman and Goldszmidt, 1998) and can even help decide the PGM structure as they naturally identify the parents of a node (Fierens et al., 2007). Relational dependency networks (RDNs) are one example of a PGM that explicitly relies on PETs (Neville and Jensen, 2007).

In the classical predictive learning setting, it is known at the time of learning which are the input and output variables, and models are constructed for this specific task. PGMs, in contrast, can predict any variable from any other variable. Motivated by this discrepancy, **multi-directional ensembles** of regression and classification trees (MERCS) have been proposed. Here, each individual tree predicts one or more variables from the other variables, and in the ensemble as a whole, every variable occurs as a target variable at least once. Essentially a non-probabilistic variant of PGMs, MERCS models have been shown to allow for much faster inference than PGMs (Van Wolputte et al., 2018), and to be useful also for missing value imputation (Van Wolputte and Blockeel, 2020).

Researchers on fuzzy logic have proposed **fuzzy decision trees** as a way of dealing with uncertain or vague data. Multiple methods for adapting decision trees to work in a fuzzy logic context have been proposed; Olaru and Wehenkel (2003) provide a good overview in their related work section. Hüllermeier and Vanderlooy (2009) argue that fuzzy decision trees are particularly advantageous for ranking, and relate this to their use of “soft” splits, where instances can be partially assigned to multiple branches.

Johansson et al. (2014) study decision trees and forests in the context of **conformal prediction**, a setting where instead of a single value, a set of values is predicted: namely, the smallest possible set for which there is a probabilistic guarantee that the true label is in it.

The above is only a selection of uses of decision trees; it is virtually impossible to be complete. While this text focuses mostly on the fields of artificial intelligence and machine learning, conceptual development of tree-based methods has happened in parallel in many different fields, including statistics and application domains such as computer vision, bioinformatics, and medical informatics; some examples are Hothorn and Lausen (2003), Strobl et al. (2007), and Criminisi et al. (2012).

## 4. Beyond recursive partitioning

Recursive partitioning is very fast, can easily be adapted to different settings (as illustrated above), and generally yields good results. Yet, other algorithms for learning decision trees have been proposed, with quite different properties. Below, we first describe adaptations of recursive partitioning to incremental and distributed learning contexts. Next, we look at how more advanced methods for searching discrete and continuous spaces have been used in decision tree learning: advanced combinatorial problem solvers, gradient descent based methods, and evolutionary algorithms.

### 4.1. Incremental learners

Incremental learners do not assume that all data is available from the beginning, but keep a preliminary model that they update when new data comes in. Such learners often use a variant of recursive partitioning that either restructures the tree when earlier choices turn out suboptimal [e.g., Utgoff (1989)], or proceeds more cautiously and splits a node only when enough data has become available in that node to be reasonably sure that this split is indeed the best choice. An example of a cautious system is VFDT (Domingos and Hulten, 2000), which uses Hoeffding bounds to guarantee with high probability that the chosen split is identical to the one that would be chosen if the whole population were looked at. The term “Hoeffding trees” is often used for trees learned this way, and there has been a wide range of follow-up work on it; see Garcia-Martin et al. (2022) for a recent contribution that includes further pointers. Also ensemble learning has been adapted to this setting; e.g., Gomes et al. (2017) learn random forests from streaming data under concept drift (where the target model may evolve over time).

### 4.2. Parallelization and distribution

By nature, decision tree computations are easy to parallelize or decentralize. This can boost runtime efficiency^4^ but also help address privacy and security concerns.

Early work on distributed learning focused on handling large, externally stored datasets; well-known examples are SLIQ (Mehta et al., 1996), SPRINT (Shafer et al., 1996), and RainForest (Gehrke et al., 2000). Later work focused on reducing communication cost in distributed implementations (e.g., Tyree et al., 2011; Meng et al., 2016a) and exploiting standard frameworks such as MapReduce (e.g., Wu et al., 2009) or Apache Spark (e.g., Meng et al., 2016b). SPDT (Ben-Haim and Tom-Tov, 2010) learns from streams in a distributed manner. Rokach (2016, Section 6.10) provides an overview with more examples.

Modern learners exploit specialized hardware, e.g., SIMD (Devos et al., 2020; Shi et al., 2022), GP-GPUs (Sharp, 2008; Wen et al., 2018), and FPGAs (Van Essen et al., 2012). Modern boosting systems such as XGBoost and LightGBM (Ke et al., 2017) all have performant GP-GPU implementations (Mitchell and Frank, 2017; Zhang et al., 2017). Not only learning, but also storage and use of decision trees is optimized, for instance using bit-level data structures, to allow deployment on edge devices with limited resources (Lucchese et al., 2017; Ye et al., 2018; Koschel et al., 2023).

Federated learning tackles the challenge of learning with data distributed among several clients. These clients collaboratively train a model under server management while keeping data decentralized (Kairouz et al., 2021), to prevent leakage of private or confidential information (Xu et al., 2021). Methods for federated learning of tree-based models rely on a variety of techniques. For instance, CryptoBoost (Jin et al., 2022) and SecureBoost (Xie et al., 2022) use *homomorphic encryption*, where data or data statistics are encrypted to allow particular computations, e.g., addition and multiplication. More generally, *secure aggregation* refers to operations and protocols that preserve information before computing impurity measures. For example, Du and Zhan (2002) proposes a scalar product protocol to compute a dot product without sharing information. *Differential privacy* provides a formal framework for privacy-preserving learning without focusing on the side of computer security. Fletcher and Islam (2019) survey and analyze differential privacy algorithms for tree-based methods.

### 4.3. Combinatorial optimization and constraint solvers

Recursive partitioning uses heuristics. Hence, it does not guarantee any kind of optimality of the resulting tree, such as being the smallest tree that perfectly fits the training data, or minimizing some loss function under a constraint on the complexity of the model. This weakness motivates the development of alternative search strategies that can provide such guarantees.

Early algorithms for finding optimal decision trees performed an exhaustive enumeration of the space of decision trees (Esmeir and Markovitch, 2007). An interesting feature of the approach of Esmeir and Markovitch (2007) is its any-time behavior: it orders the enumeration such that promising trees are enumerated first, allowing it to provide good trees if terminated early.

To obtain better run times, a number of different ideas have been explored.

An early approach was DL8 (Nijssen and Fromont, 2007), which uses results from *itemset mining* to construct provably optimal trees. At the basis of DL8 is that a branch in a decision tree can be seen as a Boolean item, and a path as a set of items. For optimization criteria such as error the optimal tree below a given path depends only on the training examples that end up at the end of the path. This makes it possible to develop algorithms that use a form of dynamic programming in which partial solutions are associated to item sets and can be reused.

To avoid finding trees that are too complex, regularizing trees can be important. The idea of regularizing the complexity of optimal decision trees, in combination with some form of dynamic programming, can also be found in OSDT (Optimal Sparse Decision Trees), proposed by Hu et al. (2019), of which an optimized version, GOSDT (Generalized and Scalable Optimal Sparse Decision Trees) was proposed by Lin et al. (2020).

Another class of methods is based on the use of mixed integer linear programming (MILP), and was pioneered by Bertsimas and Dunn (2017). Mixed Integer Linear Programming is a generic approach for solving combinatorial problems by expressing them using linear constraints over integer variables. This approach is extensible: constraints can easily be added if they can be expressed in a linear form. Follow-up work includes, for instance, an adaptation of the approach for survival trees (Bertsimas et al., 2022). While a more efficient MILP approach, BinOCT, has been proposed (Verwer and Zhang, 2019), compared to DL8 a disadvantage is that the run time performance of MILP-based approaches is not as good.

Another generic approach is based on the use of SAT solvers and Constraint Programming solvers. The use of SAT solvers was studied by Bessiere et al. (2009) and Narodytska et al. (2018), for determining whether or not for a given training data set a decision tree exists that makes no error on this training data, under a constraint on either size or depth. Avellaneda (2020) built on this approach to build an algorithm that can find the smallest depth of a consistent tree, as well as the smallest consist tree under a depth constraint.

While these SAT-based approaches focused on trees that are consistent with all training examples, on many data sets one can accept trees that make a small amount of error. Hu et al. (2020) showed that by using MaxSAT solvers instead of SAT solvers, it becomes feasible to find trees that minimize error. Constraint Programming (CP) solvers have similar benefits. The use of CP was studied by Verhaeghe et al. (2020); in this work it was shown that a DL8-style algorithm can be combined with Constraint Programming.

Aglin et al. proposed an optimized version of DL8, called DL8.5, by adding the use of branch and bound to this search algorithm (Aglin et al., 2020a) and showed how to apply this to find sparse decision trees or regression trees (Aglin et al., 2020b). Similarly, GOSDT supports other optimization criteria as well (Lin et al., 2020).

Improved bounds and data structures for DL8-style approaches were subsequently proposed by Demirovic et al. (2022). These authors report speed-ups of 1,000 and more compared to MILP-based approaches, and speed-ups of 500 and more compared to GOSDT. A shared weakness of the DL8-style algorithms is the need to store large numbers of itemsets. This weakness was studied by Aglin et al. (2022), who propose to sacrifice a certain degree of run time performance to limit memory consumption. Kiossou et al. (2022) showed how any-time behavior can be improved.

Given that most search algorithms allow for some freedom in the definition of constraints and optimization criteria, a number of them have been tuned for specific settings; these will be discussed in Section 6.

Combinations of heuristic and optimal algorithms are developed, hoping to combine the best of both worlds. The any-time algorithm of Esmeir and Markovitch (2007) is an early example of this. Another such approach was proposed by, who observe that some optimal decision tree learning algorithms require a discretization of the data and the specification of a depth constraint; they propose to learn an ensemble first using traditional greedy algorithms, and to subsequently use this ensemble to guide the choices for how to discretize the data and choose the depth constraint.

### 4.4. Gradient-based approaches

Several approaches have been proposed to learn decision trees (and forests) using gradient based approaches. The majority of these focus on oblique decision trees (where splits are based on linear combinations of input features). The key idea is to encapsulate, in a differentiable objective function, paths of instances along nodes and how the parameters (e.g., weights for the linear combination of features) affect these. Some approaches use hard (or discrete) paths computed with a threshold split at each internal node; other approaches use soft paths computed with cumulative distribution functions such as sigmoid, leading to trees called *soft* decision trees.

For the case of **hard paths**, one direction used by Norouzi et al. (2015) is to explicitly model them with discrete latent variables over internal nodes of trees, then an alternative minimization algorithm can be employed to infer paths and learn parameters of nodes using their gradients. In another direction, remarking that oblique decision trees define constant regions linearly separated in the input space, Lee and Jaakkola (2020) introduce the *locally constant networks* that are provably equivalent to oblique decision trees and are defined with derivatives of *ReLU* networks. Using locally constant networks, it is therefore possible to learn equivalent oblique decision trees with a global and differentiable objective function.

Differently from the above, in **soft decision trees**, instances are routed to each child node with a probability (Irsoy et al., 2012). Using a differentiable function such as sigmoid to compute this probability allows expressing a differentiable objective function. Examples of work that focuses on decision trees include the work of Frosst and Hinton (2018). Thanks to the success of learning soft decision trees with gradient descent, several extensions have been made for random forests. For example, the *neural decision forest* (NDF) proposed by Rota Bulo and Kontschieder (2014) is an ensemble of decision trees where split functions are randomized multi-layer perceptrons (MLPs) that are learned locally (in each decision node) using gradient descent. Another example of extension to forests is the *deep neural decision forest* (dNDF) (Kontschieder et al., 2015), which is similar to NDF except two aspects. First, a dNDF may use inside decision nodes, sigmoid units on top of deep convolutional neural networks. Second, a dNDF supports end-to-end training via gradient descent. The training of dNDF can also be sped up using dedicated activation functions (Hazimeh et al., 2020) instead of the sigmoid.

Many gradient-based approaches to tree learning assume a fixed tree structure, but some infer the tree structure as part of the learning process. This can be done by modeling the possibility for a node to be either a leaf node or a decision node, allowing therefore pruning during learning. Examples of these improvements include the *budding tree* (Irsoy et al., 2014), the one-stage tree (Xu et al., 2022) and the quadratic program of Zantedeschi et al. (2021).

The majority of gradient-based approaches focus on trees with multivariate or more complex splits (e.g., involving MLPs). This stands in contrast to the combinatorial search based methods of the previous section, which typically learn trees with univariate tests.

### 4.5. Evolutionary algorithms

Given the broad applicability of evolutionary algorithms for search, it is not surprising that such algorithms have also been used to search the space of all decision trees to find trees that fit the data well. Evolutionary search is naturally positioned between greedy search, which is fast but prone to suboptimal decisions, and exhaustive search, which gives a provably optimal solution at a high cost. Barros et al. (2012) survey the area of evolution-based decision tree learning. Among other things, they conclude that evolutionary search does frequently lead to trees with better predictive performance, which is an indication that recursive partitioning's bias toward short trees is not always advantageous. The survey also launches the idea of an evolutionary search for decision tree algorithms (rather than the tree themselves), which was followed up on in later work (Barros et al., 2015). An interesting question is how evolutionary algorithms (or evolutionarily-optimized greedy algorithms) compare to the solver-based methods mentioned before. To our knowledge, no systematic comparisons between evolutionary search and solver-based methods has been made.

## 5. Integrating constraints

An aspect of learning that has received increasing attention in recent years is how background knowledge in the use of constraints can be used in the learning process. For instance, a bank might require that its model for credit approval is monotonic in the “income” attribute: all else being equal, a client with a higher income should not get a worse score. Can we verify that a given model does not violate this constraint, or even better: can we ensure that the learner only returns models that do not violate it? We refer to the latter as imposing constraints, and to the former as model verification. There has been a substantial amount of work on both fronts, in the context of decision trees.

### 5.1. Imposing constraints on decision trees

The use of constraints is ubiquitous in machine learning due to two principal reasons. First, model regularization via constraints is a standard way to overcome the overfitting problem in several machine learning models. Second, due to the deep impact on machine learning in our society, in several critical domains (e.g., health, finance) machine learning models do not only need to provide best performance, they also need to meet several requirements such as fairness, privacy-related restrictions, consistency with prior domain knowledge, and so on. In both scenarios, constraint enforcement represents a principled framework to provide a better control of learned machine models such that these models eventually meet societal, ethical and practical goals (Cotter et al., 2019). Several works have therefore been proposed to enforce constraints on decision trees. The survey of Nanfack et al. (2021) highlights structure-level constraints, feature-level and instance-level constraints on decision trees.

Methods imposing structure-level constraints aim to learn decision trees under constraints over the structure of the tree (e.g., the size, depth). These methods may employ pruning (e.g., learn an overfitted tree through recursive partitioning and then prune this tree to reduce its size). These methods include work such as Garofalakis et al. (2003) and Struyf and Džeroski (2006). Thanks to the discrete nature of decision trees, other methods imposing structure-level constraints may also leverage combinatorial optimization to find the best accurate decision tree that satisfy the depth or size constraint (see Section 4).

Methods imposing feature-level constraints aim to learn decision trees under feature-related constraints such as monotonicity, fairness, ordering and hierarchy over selected features on the tree, and privacy. The majority of these methods use a recursive partitioning method along with constraint-aware heuristics, which help in choosing the most informative split that does not violate too much the constraint. Examples of these constraint-aware heuristics include the *total ambiguity score* of Ben-David (1995) for monotonicity constraints [see also the survey by Potharst and Feelders (2002)], the *information cost function* of Núñez (1991) for hierarchy constraints, the *fair information gain* of Zhang and Ntoutsi (2019) for fairness constraints, and the adapted exponential mechanism of Li et al. (2020) for privacy constraints. Besides, there are methods such as Aghaei et al. (2019) that use combinatorial optimization to learn decision trees under fairness constraints.

Finally, methods imposing instance-level constraints focus either on robustness constraints or on must-link and cannot-link constraints on clustering trees. Robustness is discussed in Section 6.2. Examples of works aiming to integrate must-link and cannot-link constraints include the work of Struyf and Džeroski (2006), which uses a penalized heuristic composed by two terms: the first one is the average variance instances over leaf nodes normalized by the total variance and the second one is the percentage of violated constraints.

### 5.2. Verification of decision tree ensembles

Model verification is used to assess the quality of a learned model. As such, it complements evaluating the model's performance on an unseen test set, i.e., regular *model testing*. In contrast to model testing, which by definition only considers the behavior of the model for the examples in the test set, verification considers the full domain and image of the learned function. In sensitive application domains like healthcare and air traffic control, this rigorous model evaluation is required.

Similar to formal verification of software systems, verification of machine learned models reasons about all possible inputs and their corresponding outputs, and verifies whether these input-output pairs satisfy the prescribed constraints. In practice, this typically happens by negating the given constraints, and trying to find instances that satisfy this negation. If successful, this disproves the claim that the model satisfies the prescribed constraints (and provides a counterexample); otherwise, it proves the claim.

Verification of learned models is challenging: e.g., Kantchelian et al. (2016) show that verification of tree ensembles in general is NP-hard. Despite this, there is substantial interest in it because it is widely applicable and can be used to validate a variety of questions and constraints. Some examples, each with a notable paper that has focused on this problem (not exhaustive):

- **Adversarial example generation**: can we slightly perturb example *x* so that the predicted label for this modified *x* is different? In practice, it is often the case that small imperceptible changes can be found that fool the model (Kantchelian et al., 2016).
- **Robustness checking**: does an adversarial example exist in a small neighborhood surrounding an example *x* (Chen et al., 2019b)?
- **Counterfactual example generation**: what attribute value needs to change in order to get the desired model outcome? This is similar to adversarial example generation, but usually also requires actionability and plausibility (Parmentier and Vidal, 2021).
- **Attribute importance**: can a change in one or a small set of attributes wildly affect the output of the model (Devos et al., 2021a)?
- **Fairness**: is a loan approval probability affected by race (Grari et al., 2019)?
- **Domain-specific questions**: when predicting the risk of stroke: can a person aged between 40 and 50 with a BMI less than 20 have a risk greater than 5% (Devos et al., 2021b)?
- **Safety**: are there conditions under which the model deviates more than some threshold *t* from some safe reference model? (Wei et al., 2022).

As is clear from the examples above, verification is applicable to a wide range of problems. However, ever since it was shown that neural networks (Szegedy et al., 2013) and later decision trees (Kantchelian et al., 2016) are susceptible to adversarial examples, most research has focused on adversarial example generation and robustness checking. In Section 6, we will focus on these applications in detail.

## 6. Decision trees for responsible AI

With AI increasingly affecting the lives of billions of people, there is an increased societal and academic interest in Responsible AI, by which is meant: giving due care and consideration to the consequences of using AI in certain contexts. Responsible AI implies taking measures to ensure that AI systems behave in a way that is considered fair, safe, transparent, and generally respects human rights. For machine-learned models, this often translates to ensuring that the learned models fulfill certain constraints that imply fairness, robustness, and explainability. Below, we consider each of these in turn, and discuss work in the context of decision trees.

### 6.1. Fairness

Often, a desirable characteristic of classification models is to not discriminate: given a labeled dataset and a Boolean attribute B, the model should not treat instances with property B different from the overall population. For instance, if B represents whether or not a person identifies as female, we may find it undesirable that the classifier is less likely to assign a positive label to this person.

Unfortunately, highly accurate predictive models may display such unfair behavior, as discrimination may be present in training data. This form of discrimination can not be avoided by simply removing attribute B from training data; other features may correlate with B in unexpected and complex ways. This has led to a number of different approaches that aim to balance two requirements at the same time: accuracy and *group fairness*, in which a group with a specific characteristic should be treated equally to an overall group of individuals.

The so-called “preprocessing” approaches modify the training data before the data is fed into a machine learning algorithm; these approaches can also be applied when the learning algorithm is a decision tree learning algorithm. Here, we focus on the “in-processing” approaches, that is, approaches that take into account fairness while learning a model.

A first approach was proposed by Kamiran et al. (2010). In this work, a decision tree is first learned using a heuristic that takes into account both discrimination and fairness. Subsequently, a post-processing step is used to relabel leaves to further improve a fairness score. This approach was adapted to the incremental learning setting by Zhang and Ntoutsi (2019).

While these approaches aim to find trees that represent a good trade-off between two criteria, they do not provide guarantees. Algorithms for learning optimal decision trees have been modified to provide such guarantees. Aghaei et al. (2019) adapted the MILP-solver based approach to take into account two forms of fairness; the approach optimizes a weighted sum of accuracy and fairness. Similarly, van der Linden et al. (2022) adapted the DL8-style approach such that an upper- or lower-bound on fairness can be ensured, where the fairness score proposed by Kamiran et al. (2010) is used to evaluate the fairness of a model.

Given the high predictive performance of boosted decision trees, ensuring fairness has also been studied for ensembles of decision trees. A first approach was proposed by Grari et al. (2019), in which a gradient boosting algorithm has been modified to change the gradient for instances based on the ability of an adversarial model to predict the sensitive feature from the class predicted by the ensemble model.

### 6.2. Robustness against adversarial examples

Since the discovery that tree ensembles, just like neural networks, are susceptible to adversarial examples (Kantchelian et al., 2016), much research has been devoted to the detection of robustness issues and ways to mitigate them. Before surveying it in more detail, we introduce some terminology.

For a classification problem, *x*′ is an adversarial example of a regular example *x* when it is “close to” *x*, i.e., in some neighborhood *N*(*x*) of *x*, and *f*(*x*) ≠ *f*(*x*′), with *f*(*x*) the predicted label for *x*. Generally, the assumption is made that *f* classifies *x* correctly, and that there is *truth proximity*, i.e., examples in *N*(*x*) are assumed to have the same true label (Diochnos et al., 2018). *N*(*x*) is called the **attack model**. It can be defined using a simple norm and radius ϵ, e.g. $N_{\epsilon }\left(x\right)=\left\{x^{\prime }|{\left\|x-x^{\prime }\right\|}_{\infty }<\epsilon \right\}$, but more intricate attack models also exist.^5^

Adversarial example generation is a direct application of the verification framework (see Section 5.2): given a regular example *x*, a verification tool is asked to construct an *x*′ ∈ *N*(*x*) with *f*(*x*′) ≠ *f*(*x*). If it fails, this proves the model robust with respect to *N*(*x*).

When *N*(*x*) is defined by an *l*_*p*_ norm, the smallest distance ϵ^*^ for which an adversarial example exists:


$$\begin{array}{l} \epsilon^{*}=\underset{x^{\prime }\in N\left(x\right)}{\min}{\left\|x-x^{\prime }\right\|}_{p}\;\text{such that }f\left(x\right)\neq f\left(x^{\prime }\right). \end{array}$$

can be seen as a measure for how difficult it is to attack an ensemble (Calzavara et al., 2020b).

#### 6.2.1. Verifying robustness

Various different approaches have been proposed for tree ensemble verification, varying in tooling, problem focus, and precision. Table 1 provides an overview.

**Table 1:** Overview of methods for adversarial example generation (*adv*) and robustness checking (*rob*) for tree ensembles.

**Method**	**Focus**	**Exact?**	**Anytime?**	**Generate examples?**	**Code available?**	**Supported norms**
Exact (MILP, Kantchelian et al., 2016)	rob	y	n^a^	y	y^b^	*l*_*p*_
Symbolic prediction (Kantchelian et al., 2016)	rob	y	n	y	n	*l*_0_
VeriGB (SMT, Einziger et al., 2019)	adv	y	n	y	n	*l*_∞_ ^c^
Cube attack (Andriushchenko and Hein, 2019)	adv	n	n	y	y	*l*_∞_
Merge (Chen et al., 2019b)	rob	n	y^d^	n	y	*l*_∞_
Merge+ (Wang et al., 2020)	rob	n	y^d^	n	y	*l*_*p*_
VoTE (Törnblom and Nadjm-Tehrani, 2020)	rob	y	n	y	y	*l*_*p*_
RF-ILP (ILP, Zhang et al., 2020b)	adv	y	n	y	n	*l*_0_
LT-attack (Zhang et al., 2020a)	adv	n	n	y	y	*l*_*p*_
Silva (Ranzato and Zanella, 2020)	rob	y	n	y	y	*l*_∞_
TreeCert (Calzavara et al., 2020a)	rob	n	n	y	n	*l*_*p*_ ^f^
Tree-ck (SMT, Devos et al., 2021a)	adv	y	n	y	y	*l*_*p*_ ^f^
Veritas (Devos et al., 2021b)	both	y^e^	y	y	y	*l*_∞_

The *exact* column indicates whether the returned results is guaranteed to be optimal. The *anytime* methods can be stopped at any time and will always produce bounds on the result. The supported attack model is provided in the *supported norms* column.

a A MILP solver like Gurobi [Gurobi Optimization, LLC (2022)] is anytime, but the approximate bounds are not tight enough for practical use (Devos et al., 2021b).

b Not by the original authors, but Devos et al. (2021b) and Vos and Verwer (2021) provide implementations.

c Authors claim the method works for any *p*-norm, but only evaluate *l*_∞_.

d The method is technically anytime as each *level* produces a new bound. However, the number of levels *L* is at most log_2_(*M*), with *M* the number of trees, and is set to 2 or 3 in the experiments.

e When the method is run to completion, the solution is exact.

f These systems allow a generic formulation of the attack model. For *TreeCert*, the attacker is extremely flexible and is modeled as a C program.

A first set of approaches translate the model to a mathematical formulation and use off-the-shelf solvers. Kantchelian et al. (2016) propose a mixed-integer linear programming (MILP) solution that can deal with any *l*_*p*_ norm. This was later specialized to a pure integer linear program (ILP) for binary input attributes and the *l*_0_ norm (Zhang et al., 2020b). Other have used satisfiability modulo theory (SMT): the approaches by Einziger et al. (2019), Sato et al. (2020), and Devos et al. (2021a) are similar and differ only in focus and implementation details. Calzavara et al. (2020a) and Ranzato and Zanella (2020) take inspiration from the software verification field and use *abstract interpretation*, commonly used for static program analysis, for formal verification of tree ensembles. Calzavara et al. (2022) propose a solution that verifies *resilience*, a generalization over robustness which considers all possible test sets that could be sampled.

A second set of approaches use techniques tailored to tree ensembles, rather than off–the-shelf solvers. These tend to be more efficient, but approximate. Chen et al. (2019b) reformulate the verification task as a maximum-clique problem in an *M*-partite graph, with *M* the number of trees. Wang et al. (2020) extend Chen et al. (2019b), which only supports the *l*_∞_ norm, to any *l*_*p*_ norm, *p* ∈ [0, ∞]. These two approaches are fast, but only produce a coarse lower bound on the robustness value, and do not generate adversarial examples. These issues are resolved by Devos et al. (2021b), who propose a heuristic search in the same graph representation that can generate concrete examples and produces anytime lower and upper bounds on the ensemble's output. Zhang et al. (2020a) use the concept of neighboring cliques (they call it *leaf tuples*) in an efficient greedy search procedure that only changes one component of the clique per step. They focus on adversarial example generation instead of robustness checking.

The MILP approach by Kantchelian et al. (2016) is the most frequently used baseline and evaluation tool for robust tree methods in the literature (see Section 6.2.2). It produces exact results within a reasonable time frame. The other exact approaches using SMT are less efficient. It is unclear how well the methods based on abstract interpretation perform in practice, as they are only evaluated on smaller datasets. The same is true for the method of Törnblom and Nadjm-Tehrani (2020).

In the authors' experience, Zhang et al. (2020a) and Devos et al. (2021b) offer the best tradeoff between accuracy and efficiency for adversarial example generation, whereas for robustness checking, Kantchelian et al. (2016) and Devos et al. (2021b) are recommended.

#### 6.2.2. Improving robustness

From the multitude of papers and methods in the previous section, it is clear that decision tree ensembles are not robust. Hence, researchers have investigated making decision trees and their ensembles more robust. Table 2 gives an overview of the available methods [extends the overviews by Vos and Verwer (2021) and Guo et al. (2022)].

**Table 2:** Overview of methods for robust decision tree learning.

**Method**	**Ensemble**	**Complexity**	**Norm**	**Guarantees?**	**Code available?**
Adversarial boosting (Kantchelian et al., 2016)	GB	*n*log(*n*)	*l*_0_	n	n
RobustTrees (Chen et al., 2019a)	RF+GB	*n*log(*n*)	*l*_∞_	n	y
RobustStumps (Andriushchenko and Hein, 2019)	GB	*n* ^2^	*l*_∞_	y	y
TREANT (Calzavara et al., 2020b)	RF	*n* ^2^	*l*_*p*_ ^a^	y	y^b^
MetaSilvae (Ranzato and Zanella, 2021)	RF	?^c^	*l*_∞_	n	y
Feat. Part. Forests (Calzavara et al., 2021)	RF	*n*log(*n*)	*l*_0_	y	n
GROOT (Vos and Verwer, 2021)	RF	*n*log(*n*)	*l*_∞_	n	y
CostAwareRobust (Chen et al., 2021)	RF+GB	*n*log(*n*)	*l*_∞_ ^d^	n	y
ROCT (Vos and Verwer, 2022)	Single	exp(*n*)	*l*_∞_ ^d^	y	y
Relabeling (Vos and Verwer, 2023)	RF+GB	*n* ^2.5^	*l*_∞_ ^e^	y	y
FPRDT (Guo et al., 2022)	Single	*n*log(*n*)	*l*_∞_	y	n
PRAdaBoost (Guo et al., 2022)	Ada	*n*log(*n*)	*l*_∞_	y	n

Some methods robustify a single tree (*Single*), and others are used in ensembles: random forest (*RF*), gradient boosting (*GB*) or AdaBoost (*Ada*). This is indicated in the *Ensemble* column. The *Complexity* column shows the complexity of the learning algorithm in number of examples *n*. The number of features and the size of the models is ignored. The *Norm* column lists the attack model that is considered by the method. The *Guarantees* column has a yes (*y*) value when the learned models are guaranteed to be robust.

a TREANT has a flexible attack model in the form of rewriting rules, allowing asymmetric perturbations (e.g. only positive), and a maximum budget (e.g. an *l*_1_-norm).

b Vos and Verwer (2021) provide an alternative implementation.

c The paper includes experiments that show that the genetic algorithm converges in 50-70 iterations for the tested datasets.

d An asymmetric attack model is supported, i.e., it is possible to allow larger positive than negative perturbations, but it is still a box constraint.

e Other norms are possible, but this is not evaluated.

In general, robust training can be formulated as the following min-max problem (Madry et al., 2018), with attack model *N*(*x*), loss function *l*, training examples (*x*_*i*_, *y*_*i*_), and ensemble *f*:


(1) $$\begin{array}{l} f^{*}=\underset{f}{\mathrm{argmin}}\left\{\overset{N}{\underset{i=1}{\sum }}\underset{x^{\prime }\in N\left(x_{i}\right)}{\max}l\left(f\left(x^{\prime }\right),y_{i}\right)\right\} \end{array}$$

The outer minimization is the usual learning optimization problem minimizing the loss function. The inner maximization models the worst-case scenario where an adversary attempts to maximize the loss of the model (e.g., flip the label) in the neighborhood of a training example *x*.

The problem is tackled from multiple different angles. Kantchelian et al. (2016) propose enriching the training data with adversarial examples, as has been done before for neural networks (Szegedy et al., 2013). Vos and Verwer (2022) use the ideas from optimal trees (see Section 4) to learn robust trees. Ranzato and Zanella (2021) use a genetic algorithm to learn robust trees. A number of papers change the splitting procedure used during the construction of the trees. The main idea is to avoid splitting thresholds that lie in dense areas; examples with values close to those thresholds can easily jump to the other side with a small perturbation. Others look at the global loss, assume a tree structure or a specific loss function, and rewrite Equation 1 to simplify the problem. Lastly, there are two approaches that are orthogonal to the previous methods. The first is a pre-processing procedure that partitions the features between the trees in the ensemble in such a way that it becomes impossible to ever trick the majority of the trees (Calzavara et al., 2021). The second is a post-processing procedure that relabels the leaves of the ensemble to make it more difficult to find neighboring leaves that predict different classes (Vos and Verwer, 2023).

Chen et al. (2019a), Vos and Verwer (2021), and Chen et al. (2021) propose changes to the splitting procedure. Chen et al. (2019a) consider the *ambiguity set* of examples that can flip sides with an ϵ perturbation, and propose a combinatorial optimization problem that finds the configuration of the examples in the ambiguity set that maximally worsens the loss (the maximization in Equation 1). This combinatorial problem cannot be practically solved, so both Chen et al. (2019a) and Chen et al. (2021) introduce approximations. Vos and Verwer (2021) improve upon this work by proposing an exact analytical solution for the Gini impurity. The result is a scalable, robust decision tree learner called GROOT. The method proposed by Calzavara et al. (2020b) is similar, but solves the combinatorial problem as a convex numerical optimization problem. Additionally, they assure global robustness by introducing the *attack invariance* property, which keeps track of the attack surface across different leaves in the tree.

Andriushchenko and Hein (2019) and Guo et al. (2022) look at the global loss of Equation 1. Andriushchenko and Hein (2019) limits the weak learners to decision stumps (trees with a single split at the root and two leaves). This allows them to split up the problem into one independent problem per attribute. Guo et al. (2022) consider the 0/1 loss and realize that this loss can be used directly to evaluate split candidates in constant time. Ranzato and Zanella (2021), Vos and Verwer (2022), and Guo et al. (2022) all use variants of *robust* 0/1 loss, where an example *x* is only considered correctly classified when all instances in *N*(*x*) receive the same label.

### 6.3. Explainability

In critical domains such as finance and health, the adoption of machine learning models may require trustworthy guarantees such as transparency.^6^ In addition to giving accurate predictions, machine learning models then must provide explanations for their predictions in human-understandable terms (Ribeiro et al., 2016a; Doshi-Velez and Kim, 2017). This motivates the research area called eXplainable Artificial Intelligence (XAI). In XAI, there are several types of explanations, including decision rules, visualization, variable importance, and counterfactual explanations. Below, we describe how decision trees can play a role in this.

#### 6.3.1. Decision tree explanations

By nature, decision trees can explain their predictions using decision rules of the form “decision *D* was made because condition *C* was fulfilled”. The explanatory power of decision tree models can be further improved by constraining its complexity (e.g., find the maximally accurate tree of depth at most *d*, see also Section 4) or by explicating relevant properties, such as which features are most important and how features interact (e.g., Lundberg et al., 2019).

Given these desirable properties, methods have been designed to approximate black-box models (or parts of them) with decision trees, so that they inherit to some extent these properties.

The most classical approach where decision trees are used is **knowledge distillation**. In knowledge distillation, decision trees *f* are trained to approximate the black-box model *g* either *locally*, in the neighborhood of an instance *x* ($\underset{f,x^{\prime }\in N\left(x\right)}{\min}d(f\left(x^{\prime }\right),g\left(x^{\prime }\right))+\Omega \left(f\right)$), or *globally* ($\underset{f}{\min}d(f,g)+\Omega \left(f\right)$), where Ω(*f*) is the complexity of the decision tree *f*. For local explainability, a well-known method is LIME (Ribeiro et al., 2016b). In LIME, decision trees can be used as the interpretable model that locally approximates the black-box model. For global explainability, examples of methods that use decision trees include TREPAN (Craven and Shavlik, 1995), its improved version TREPAN Reloaded (Confalonieri et al., 2020) and the soft distilled decision tree of Frosst and Hinton (2018). In knowledge distillation, the standard setup is to use the empirical distribution to approximate the black-box model. However, for a better approximation, the work of Bastani et al. (2017) distills random forests on a decision tree using samples from a fitted mixture of truncated normal distributions.

Methods using the knowledge distillation approach may not work well with high-dimensional data such as image and text data because interpretable univariate trees may not be suitable for this type of data. That is why several methods have been proposed to design new models using or having decision trees as **interpretable components**. For example, Wu et al. (2018) and Okajima and Sadamasa (2019) respectively use decision trees and extracted decision rules to constrain deep neural networks for improved interpretability. Other methods such as the neural prototype trees (ProtoTrees) (Nauta et al., 2021) and recurrent decision tree models (Alaniz et al., 2021) integrate decision trees in fully differentiable models with convolutional neural networks and recurrent neural networks, respectively. Besides this, there is a considerable literature on neural tree approaches (see the dNDF in Section 4.4) where the goal is to combine neural networks and decision trees models with the ambition to get the advantages of the two models: interpretability without sacrificing accuracy. The recent survey of Li et al. (2022) analyses the majority of this work.

#### 6.3.2. Counterfactual explanations of decision trees and forests

In the previous section, through decision rule explanations, decision trees were mainly described to provide explanations of black-box models. This section focuses on a different type of explanations, counterfactual explanations, which may be used to locally explain soft decision trees and tree ensembles.

Counterfactual explanations aim to provide an answer to questions of the type “what should I have done differently to get a different outcome?”. They provide minimal changes that can be performed on input features of an instance *x* to change its prediction *f*(*x*). More formally, in the simplest setting, a counterfactual explanation is an instance *x*′ such that (1) the prediction of *x*′ differs from *f*(*x*), i.e., *f*(*x*′) ≠ *f*(*x*), (2) *x* and *x*′ are close under a metric, (3) *x*′ is a plausible input (Albini et al., 2022), where plausible may mean a realistic instance that lies in the data manifold. Apart from the requirement of plausibility, counterfactual explanations are closely related to adversarial examples (described in Section 5.2) and their generation can be framed as a constrained optimization problem. Since univariate decision trees are interpretable by design (through decision rules), there is little interest to provide counterfactual explanations on them. However, there is a growing interest in designing methods that are able to provide this type of explanations for oblique decision trees and tree ensembles.

Although there exist several agnostic (that do not depend on the model class) methods to generate counterfactual explanations [see the recent survey of Guidotti (2022)], few of them apply to (oblique) decision trees because of the non-differentiability. This motivated Carreira-Perpiñán and Hada (2021) to propose a closed-form solution (resp. quadratic program) for univariate decision trees (resp. oblique decision trees) to find counterfactual explanations.

On tree ensemble models, Cui et al. (2015) showed that the constrained optimization problem of generating counterfactual explanations is NP-hard. Therefore, through the lens of optimization, there are heuristic based approaches and optimal based approaches to generate counterfactual explanations for tree ensemble models.

Tolomei et al. (2017) propose a heuristic method that breaks the computational complexity by searching counterfactual explanations on only at least half of decision trees (in the ensemble) that give the desired outcome.

The majority of optimality based approaches leverage MILP solvers to model the generation of *optimal* counterfactual explanations for a tree ensemble. Among the earliest work in this direction is the work of Cui et al. (2015). While their framework was general enough to cope with all *l*_*p*_ norms, Cui et al. (2015) eventually consider only the Mahalanobis distance and use a discretization in the input space to permit a modeling (of the MILP problem) with only integer variables. Still using integer variables, this framework has been recently improved by Kanamori et al. (2021), extending it to an *l*_1_ norm. Remarking that integer variables usually slow down the optimization done by the MILP solver (due to their implication in the branch and bound), Parmentier and Vidal (2021) recently introduce a new MILP formulation that significantly reduces the number of integer variables. As a result, their formulation allows to generate optimal counterfactual explanations in seconds for moderated-size problems (hundreds of trees and over fifty features).

The different types of approaches among tree learners (see Section 4) are reflected also here; for instance, Lucic et al. (2022) show how methods originally proposed for differentiable models can be used with tree ensembles.

A significant issue with counterfactual examples for trees and ensembles is their robustness to changes in the model: an example that is counterfactual for a model may no longer be if the model is retrained on slightly different data. Dutta et al. (2022) study how to generate robust counterfactual examples.

## 7. Challenges and perspectives

It stands out from the preceding sections that there is a recent resurgence in the use of decision trees. Their discrete nature readily allows (1) the extraction of human-readable decision rules and (2) the full verification of the input-output mapping defined by the trees. This stands in stark contrast to (deep) neural networks. While the performance of deep learning is unchallenged on many tasks, extracting human-interpretable information about the network is much more challenging and, as it stands now, verification of neural networks seems to be more difficult to scale to realistic problem scenarios than verification of tree ensembles. For an overview of verification in deep neural networks, see Liu et al. (2021).

The aforementioned reasons explain why many approaches are again considering decision trees, either by themselves, or as surrogate models. For example, decision trees are often the target models in knowledge distillation for interpretability and explanations (Ribeiro et al., 2016b; Confalonieri et al., 2020) (see Section 6.3), and they are used in reinforcement learning for policy verification (Bastani et al., 2018; Milani et al., 2022). While neural networks are particularly well-suited to reinforcement learning given their natural ability to continuously update the weights, we see that decision trees are used again purely for their interpretability and ease of verification, even when that means giving up some performance.

As discussed in Section 6.2, robustness is a major open challenge in decision tree ensembles, and the field of robust trees is growing rapidly, with multiple dimensions being explored simultaneously. A first important issue arises because most tree ensembles are non-continuous, non-smooth step functions, which means it is not straightforward to reason about the smoothness of the function. Coincidentally, robustness and smoothness are linked (Yang et al., 2020): a model that is non-robust for an instance *x*, i.e., a perturbed example *x*′ close to *x* exists with a different prediction, must inevitable have a large rate of change in output between *x* and *x*′. Tree ensembles predict constant values for discrete subsections of the input space. There is no smoothness constraint between values predicted for neighboring subsections. Contrast this to smooth continuous functions where assumptions can be made about the rate of change between close points in the input space. A second important issue is due to the fact that the number of attributes tested to reach a leaf is limited to the depth of the leaf in the tree. Assuming that the number of attributes in the data is relatively large, many of the attributes are unconstrained given the prediction of a particular tree. In an ensemble, the next tree is likely going to pick different attributes. Correlations exist in the data distribution, but the trees do not strictly enforce them. After all, a split on a strongly correlated feature is unlikely to yield a better partitioning of the data. An attacker can exploit this as follows. Making small perturbations in one attribute might flip a split in one tree, but will not affect another tree that happened to split on a correlated attribute. An attack can use this to carefully select the branches in the trees to attain a desired outcome.

Section 6 showed challenges tackled with tree-based models in the context of responsible AI. These issues, which include fairness, robustness and explainability, are mainly addressed *in isolation* in the literature, although they are clearly related (see, e.g., the analogy between counterfactual explanations and adversarial example generation in Sections 5.2 and 6.3.2). There are very few studies that link all these components in a single framework or that thoroughly investigate the possible (in)compatibility of the requirements of responsible and trustworthy AI. It is expected that future studies will fill this gap, in particular for tree-based methods.

## 8. Concluding remarks

Decision trees have been a cornerstone of machine learning from its very beginning, and will likely remain so for decades to come. Some reasons for this are:

1. Predictive performance: Tree ensembles have unrivaled predictive accuracy when learning from tabular data.
2. Efficiency: Trees can be learned from relatively small amounts of data. Learning is very fast, prediction extremely fast; this is useful especially when deploying models on mobile devices.
3. Ease of use: Good results can often be obtained without hyperparameter tuning (though tuning may further improve them).
4. Interpretability: Individual predictions are easy to interpret.
5. Flexibility: Tree learning algorithms are easily adapted for tasks beyond the usual classification and regression.
6. Versatility: Decision trees can be used for a wide variety of tasks, ranging well beyond classification and regression.
7. Suitability for auxiliary use: Decision trees are often useful as auxiliary models, and are easily integrated in other systems.
8. Verifiability: The structure of trees and forests are such that they can be subject to formal verification.
9. Constrainability: Structural and semantic constraints can be imposed on trees and ensembles.

Properties 1–4 explain the continued popularity of decision trees for predictive modeling. Properties 5–9 make decision trees and forests very useful in the context of responsible AI: they facilitate the development of AI systems that are accurate, robust, fair, and transparent. It seems likely that decision tree based models will continue to be useful when other challenges arise in AI, and that research on decision trees (both how to learn them, and how to use them) will remain relevant in the future.

Research directions hitherto little explored include: (a) extending the “optimal tree learning” methods to dynamic settings (incremental learning, concept drift), ensembles, and variants of decision trees (oblique trees, multi-target trees, ranking, predictive clustering trees, etc.); (b) imposing and/or verifying a broader variety of constraints on trees and ensembles; (c) exploiting the commonalities between domains currently mostly studied in isolation, such as robust and explanatory AI; (d) cross-comparing methods from different paradigms (such as combinatorial solvers versus evolutionary approaches). Further developments in the area of responsible AI will likely keep exploiting existing decision tree technologies as well as motivate new research.

## Author contributions

All authors listed have made a substantial, direct, and intellectual contribution to the work and approved it for publication.

## Footnotes

^1^E.g., when only three instances remain, any binary test that on the population does not correlate with the class labels still has a probability of 1/4 of yielding a perfect split.

^2^See, e.g., the Kaggle platform for machine learning competitions.

^3^Tuning may still be helpful, but using standard settings typically already gives good results.

^4^In this text, the term efficiency typically refers to computational efficiency; we explicitly write *runtime efficiency* and *memory efficiency* in other cases.

^5^E.g., some papers use asymmetric attack models where different perturbation sizes are used in either direction (Törnblom and Nadjm-Tehrani, 2020; Devos et al., 2021b; Vos and Verwer, 2021). Others add a maximum budget (Devos et al., 2021a) and others again use rewriting rules, which corresponds to a conditional asymmetric model with a maximum budget (Calzavara et al., 2020b).

^6^Communication from the Commission of 8 April 2019, Ethics Guidelines for Trustworthy AI, COM (2019).

## References

[B1] Aghaei S. Azizi M. J. Vayanos P. (2019). Learning optimal and fair decision trees for non-discriminative decision-making, in Proceedings of the 33rd AAAI Conference on Artificial Intelligence 1418–1426. 10.1609/aaai.v33i01.33011418

[B2] Aglin G. Nijssen S. Schaus P. (2020a). Learning optimal decision trees using caching branch-and-bound search, in Proceedings of the 34th AAAI Conference on Artificial Intelligence 3146–3153. 10.1609/aaai.v34i04.5711

[B3] Aglin G. Nijssen S. Schaus P. (2020b). Pydl8.5: a library for learning optimal decision trees, in Proceedings of the 29th International Joint Conference on Artificial Intelligence 5222–5224. 10.24963/ijcai.2020/750

[B4] Aglin G. Nijssen S. Schaus P. (2022). Learning optimal decision trees under memory constraints, in Machine Learning and Knowledge Discovery in Databases (ECMLPKDD 2022), Part V 393–409. 10.1007/978-3-031-26419-1_24

[B5] Alaniz S. Marcos D. Schiele B. Akata Z. (2021). Learning decision trees recurrently through communication, in Proceedings of the IEEE/CVF Conference on Computer Vision and Pattern Recognition 13518–13527. 10.1109/CVPR46437.2021.01331

[B6] Albini E. Long J. Dervovic D. Magazzeni D. (2022). Counterfactual shapley additive explanations, in 2022 ACM Conference on Fairness, Accountability, and Transparency 1054–1070. 10.1145/3531146.3533168

[B7] Andriushchenko M. Hein M. (2019). Provably robust boosted decision stumps and trees against adversarial attacks, in Advances in Neural Information Processing Systems 12997–13008.

[B8] Avellaneda F. (2020). Efficient inference of optimal decision trees, in Proceedings of the 34th AAAI Conference on Artificial Intelligence 3195–3202. 10.1609/aaai.v34i04.5717

[B9] Barros R. C. Basgalupp M. P. de Carvalho A. C. P. L. F. Freitas A. A. (2012). A survey of evolutionary algorithms for decision-tree induction. IEEE Trans. Syst. Man, Cyber. 42, 291–312. 10.1109/TSMCC.2011.215749411079905

[B10] Barros R. C. de Carvalho A. C. P. L. F. Freitas A. A. (2015). Automatic Design of Decision-Tree Induction Algorithms. New York: Springer Briefs in Computer Science. 10.1007/978-3-319-14231-9

[B11] Bastani O. Kim C. Bastani H. (2017). Interpretability via model extraction. arXiv:1706.09773.

[B12] Bastani O. Pu Y. Solar-Lezama A. (2018). Verifiable reinforcement learning via policy extraction, in Advances in Neural Information Processing Systems 2499–2509. 33690619

[B13] Bekker J. Davis J. (2018). Estimating the class prior in positive and unlabeled data through decision tree induction, in Proceedings of the 32nd AAAI Conference on Artificial Intelligence 2712–2719. 10.1609/aaai.v32i1.11715

[B14] Ben-David A. (1995). Monotonicity maintenance in information-theoretic machine learning algorithms. Mach. Learn. 19, 29–43. 10.1007/BF00994659

[B15] Ben-Haim Y. Tom-Tov E. (2010). A streaming parallel decision tree algorithm. J. Mach. Lear. Res. 11, 849–872.

[B16] Bertsimas D. Dunn J. (2017). Optimal classification trees. Mach. Lear. 106, 1039–1082. 10.1007/s10994-017-5633-9

[B17] Bertsimas D. Dunn J. Gibson E. Orfanoudaki A. (2022). Optimal survival trees. Mach. Lear. 111, 2951–3023. 10.1007/s10994-021-06117-0

[B18] Bessiere C. Hebrard E. O'Sullivan B. (2009). Minimising decision tree size as combinatorial optimisation, in Principles and Practice of Constraint Programming - CP 2009 173–187. 10.1007/978-3-642-04244-7_16

[B19] Blockeel H. De Raedt L. (1998). Top-down induction of first-order logical decision trees. Artif. Intell. 101, 285–297. 10.1016/S0004-3702(98)00034-4

[B20] Blockeel H. Page D. Srinivasan A. (2005). Multi-instance tree learning, in Proceedings of the 22nd International Conference on Machine Learning 57–64. 10.1145/1102351.110235936673169

[B21] Blockeel H. Raedt L. D. Ramon J. (1998). Top-down induction of clustering trees, in Proceedings of the 15th International Conference on Machine Learning 55–63. 15460282

[B22] Bou-Hamad I. Larocque D. Ben-Ameur H. (2011). A review of survival trees. Stat. Surv. 5, 44–71. 10.1214/09-SS047

[B23] Breiman L. (1996). Bagging predictors. Mach. Lear. 24, 123–140. 10.1007/BF00058655

[B24] Breiman L. (2001). Random forests. Mach. Lear. 45, 5–32. 10.1023/A:1010933404324

[B25] Breiman L. Friedman J. H. Olshen R. A. Stone C. J. (1984). Classification and Regression Trees. Washington, DC: Wadsworth.

[B26] Calzavara S. Cazzaro L. Lucchese C. Marcuzzi F. Orlando S. (2022). Beyond robustness: Resilience verification of tree-based classifiers. Comput. Secur. 121, 102843. 10.1016/j.cose.2022.102843

[B27] Calzavara S. Ferrara P. Lucchese C. (2020a). Certifying decision trees against evasion attacks by program analysis, in Computer Security-ESORICS 2020: 25th European Symposium on Research in Computer Security 421–438. 10.1007/978-3-030-59013-0_21

[B28] Calzavara S. Lucchese C. Marcuzzi F. Orlando S. (2021). Feature partitioning for robust tree ensembles and their certification in adversarial scenarios. EURASIP J. Inform. Secur. 2021, 1–17. 10.1186/s13635-021-00127-0

[B29] Calzavara S. Lucchese C. Tolomei G. Abebe S. A. Orlando S. (2020b). Treant: training evasion-aware decision trees. Data Mining Knowl. Discov.34, 1390–1420. 10.1007/s10618-020-00694-9

[B30] Carreira-Perpi nán M. Á. Hada S. S. (2021). Counterfactual explanations for oblique decision trees: Exact, efficient algorithms, in Proceedings of the 35th AAAI Conference on Artificial Intelligence 6903–6911. 10.1609/aaai.v35i8.16851

[B31] Chen H. Zhang H. Boning D. Hsieh C.-J. (2019a). Robust decision trees against adversarial examples, in Proceedings of the 36th International Conference on Machine Learning 1122–1131.

[B32] Chen H. Zhang H. Si S. Li Y. Boning D. Hsieh C.-J. (2019b). Robustness verification of tree-based models, in Advances in Neural Information Processing Systems 12317–12328.

[B33] Chen T. Guestrin C. (2016). Xgboost: A scalable tree boosting system, in Proceedings of the 22nd ACM SIGKDD International Conference on Knowledge Discovery and Data Mining 785–794. 10.1145/2939672.2939785

[B34] Chen Y. Wang S. Jiang W. Cidon A. Jana S. (2021). Cost-aware robust tree ensembles for security applications, in 30th USENIX Security Symposium (USENIX Security 21) 2291–2308.

[B35] Clémençon S. Depecker M. Vayatis N. (2011). Adaptive partitioning schemes for bipartite ranking. Mach. Lear. 83, 31–69. 10.1007/s10994-010-5190-y

[B36] Confalonieri R. Weyde T. Besold T. R. del Prado Martín F. M. (2020). Trepan reloaded: A knowledge-driven approach to explaining artificial neural networks, in 24th European Conference on Artificial Intelligence 2457–2464.

[B37] Costa V. Pedreira C. (2023). Recent advances in decision trees: an updated survey. Artif. Intell. Rev. 56, 4765–4800. 10.1007/s10462-022-10275-5

[B38] Cotter A. Jiang H. Gupta M. R. Wang S. Narayan T. You S.. (2019). Optimization with non-differentiable constraints with applications to fairness, recall, churn, and other goals. J. Mach. Lear. Res. 20, 1–59.

[B39] Craven M. W. Shavlik J. W. (1995). Extracting tree-structured representations of trained networks, in Proceedings of the 8th International Conference on Neural Information Processing Systems 24–30.

[B40] Criminisi A. Shotton J. (2013). Decision Forests for Computer Vision and Medical Image Analysis. Berlin: Springer Publishing Company, Incorporated. 10.1007/978-1-4471-4929-3

[B41] Criminisi A. Shotton J. Konukoglu E. (2012). Decision forests: A unified framework for classification, regression, density estimation, manifold learning and semi-supervised learning. Found. Trends Comput. Graph. Vision 7, 81–227. 10.1561/0600000035

[B42] Cui Z. Chen W. He Y. Chen Y. (2015). Optimal action extraction for random forests and boosted trees, in Proceedings of the 21th ACM SIGKDD International Conference on Knowledge Discovery and Data Mining 179–188. 10.1145/2783258.2783281

[B43] Demirovic E. Lukina A. Hebrard E. Chan J. Bailey J. Leckie C.. (2022). Murtree: Optimal decision trees via dynamic programming and search. J. Mach. Lear. Res. 23, 1–26.

[B44] Devos L. Meert W. Davis J. (2020). Fast gradient boosting decision trees with bit-level data structures, in Machine Learning and Knowledge Discovery in Databases (ECMLPKDD 2019), Part I 590–606. 10.1007/978-3-030-46150-8_35

[B45] Devos L. Meert W. Davis J. (2021a). Verifying tree ensembles by reasoning about potential instances, in Proceedings of the 2021 SIAM International Conference on Data Mining 450–458. 10.1137/1.9781611976700.51

[B46] Devos L. Meert W. Davis J. (2021b). Versatile verification of tree ensembles, in Proceedings of the 38th International Conference on Machine Learning 2654–2664.

[B47] Diochnos D. Mahloujifar S. Mahmoody M. (2018). Adversarial risk and robustness: General definitions and implications for the uniform distribution, in Advances in Neural Information Processing Systems 10380–10389.

[B48] Domingos P. M. Hulten G. (2000). Mining high-speed data streams, in Proceedings of the 6th ACM SIGKDD International Conference on Knowledge Discovery and Data Mining 71–80. 10.1145/347090.347107

[B49] Dong X. Yu Z. Cao W. Shi Y. Ma Q. (2020). A survey on ensemble learning. Front Comput. Sci. 14, 241–258. 10.1007/s11704-019-8208-z

[B50] Doshi-Velez F. Kim B. (2017). Towards a rigorous science of interpretable machine learning. arXiv:1702.08608.

[B51] Du W. Zhan Z. (2002). Building decision tree classifier on private data, in Proceedings of the 14th IEEE International Conference on Privacy, Security and Data Mining 1–8.

[B52] Dutta S. Long J. Mishra S. Tilli C. Magazzeni D. (2022). Robust counterfactual explanations for tree-based ensembles, in Proceedings of the 39th International Conference on Machine Learning 5742–5756.

[B53] Einziger G. Goldstein M. Sa'ar Y. Segall I. (2019). Verifying robustness of gradient boosted models, in Proceedings of the 33rd AAAI Conference on Artificial Intelligence 2446–2453. 10.1609/aaai.v33i01.33012446

[B54] Esmeir S. Markovitch S. (2007). Anytime learning of decision trees. J. Mach. Lear. Res. 8, 891–933.

[B55] Fierens D. Ramon J. Blockeel H. Bruynooghe M. (2010). A comparison of pruning criteria for probability trees. Mach. Lear. 78, 251–285. 10.1007/s10994-009-5147-1

[B56] Fierens D. Ramon J. Bruynooghe M. Blockeel H. (2007). Learning directed probabilistic logical models: Ordering-search versus structure-search, in Proceedings of the 18th European Conference on Machine Learning 567–574. 10.1007/978-3-540-74958-5_54

[B57] Fletcher S. Islam M. Z. (2019). Decision tree classification with differential privacy: A survey. ACM Comput. Surv. 52, 1–33. 10.1145/3337064

[B58] Freund Y. Mason L. (1999). The alternating decision tree learning algorithm, in Proceedings of the 16th International Conference on Machine Learning 124–133.

[B59] Friedman J. H. (2001). Greedy function approximation: A gradient boosting machine. Ann. Stat. 29, 1189–1232. 10.1214/aos/1013203451

[B60] Friedman N. Goldszmidt M. (1998). Learning bayesian networks with local structure, in Learning in Graphical Models (Springer) 421–459. 10.1007/978-94-011-5014-9_15

[B61] Frosst N. Hinton G. (2018). Distilling a neural network into a soft decision tree, in Proceedings of the First International Workshop on Comprehensibility and Explanation in AI and ML. 33286971

[B62] Fürnkranz J. Hüllermeier E. (2010). Preference Learning. Berlin: Springer.

[B63] Garcia-Martin E. Bifet A. Lavesson N. König R. Linusson H. (2022). Green accelerated Hoeffding tree. arXiv:2205.03184.

[B64] Garofalakis M. Hyun D. Rastogi R. Shim K. (2003). Building decision trees with constraints. Data Min. Knowl. Disc. 7, 187–214. 10.1023/A:1022445500761

[B65] Gehrke J. Ramakrishnan R. Ganti V. (2000). Rainforest-a framework for fast decision tree construction of large datasets. Data Min. Knowl. Disc. 4, 127–162. 10.1023/A:100983982979321523931

[B66] Gomes H. M. Bifet A. Read J. Barddal J. P. Enembreck F. Pfharinger B.. (2017). Adaptive random forests for evolving data stream classification. Mach. Lear. 106, 1469–1495. 10.1007/s10994-017-5642-833266741

[B67] Grari V. Ruf B. Lamprier S. Detyniecki M. (2019). Fair adversarial gradient tree boosting, in Proceedings of the 2019 IEEE International Conference on Data Mining 1060–1065. 10.1109/ICDM.2019.00124

[B68] Grinsztajn L. Oyallon E. Varoquaux G. (2022). Why do tree-based models still outperform deep learning on typical tabular data? in NeurIPS 2022 Datasets and Benchmarks.

[B69] Guidotti R. (2022). Counterfactual explanations and how to find them: literature review and benchmarking. Data Min. Knowl. Disc. 3, 1–55. 10.1007/s10618-022-00831-6

[B70] Guo J.-Q. Teng M.-Z. Gao W. Zhou Z.-H. (2022). Fast provably robust decision trees and boosting, in Proceedings of the 39th International Conference on Machine Learning 8127–8144.

[B71] Gurobi Optimization L. L. C. (2022). Gurobi Optimizer Reference Manual.

[B72] Hazimeh H. Ponomareva N. Mol P. Tan Z. Mazumder R. (2020). The tree ensemble layer: Differentiability meets conditional computation, in Proceedings of the 37th International Conference on Machine Learning 4138–4148.

[B73] Hothorn T. Lausen B. (2003). Bagging tree classifiers for laser scanning images: a data- and simulation-based strategy. Artif. Intell. Med. 27, 65–79. 10.1016/S0933-3657(02)00085-4 12473392

[B74] Hu H. Siala M. Hebrard E. Huguet M.-J. (2020). Learning optimal decision trees with maxsat and its integration in adaboost, in Proceedings of the 29th International Joint Conference on Artificial Intelligence 1170–1176. 10.24963/ijcai.2020/163

[B75] Hu X. Rudin C. Seltzer M. I. (2019). Optimal sparse decision trees, in Advances in Neural Information Processing Systems 7265–7273. 37786624 PMC10544768

[B76] Hüllermeier E. Vanderlooy S. (2009). Why fuzzy decision trees are good rankers. IEEE Trans. Fuzzy Syst. 17, 1233–1244. 10.1109/TFUZZ.2009.2026640

[B77] Hyafil L. Rivest R. (1976). Constructing optimal binary decision trees is np-complete. Inform. Proces. Lett. 5, 15–17. 10.1016/0020-0190(76)90095-8

[B78] Irsoy O. Yıldız O. T. Alpaydın E. (2012). Soft decision trees, in Proceedings of the 21st International Conference on Pattern Recognition 1819–1822.

[B79] Irsoy O. Yildiz O. T. Alpaydin E. (2014). Budding trees, in Proceedings of the 22nd International Conference on Pattern Recognition 3582–3587. 10.1109/ICPR.2014.616

[B80] Jin C. Wang J. Teo S. G. Zhang L. Chan C. Hou Q.. (2022). Towards end-to-end secure and efficient federated learning for xgboost, in Proceedings of the AAAI International Workshop on Trustable, Verifiable and Auditable Federated Learning.

[B81] Johansson U. Boström H. Löfström T. Linusson H. (2014). Regression conformal prediction with random forests. Mach. Lear. 97, 155–176. 10.1007/s10994-014-5453-0

[B82] Kairouz P. McMahan H. B. Avent B. Bellet A. Bennis M. Nitin Bhagoji A.. (2021). Advances and open problems in federated learning. Found. Trends Mach. Learn. 14, 1–210. 10.1561/2200000083

[B83] Kamiran F. Calders T. Pechenizkiy M. (2010). Discrimination aware decision tree learning, in Proceedings of the 10th IEEE International Conference on Data Mining 869–874. 10.1109/ICDM.2010.5030424467

[B84] Kanamori K. Takagi T. Kobayashi K. Arimura H. (2021). Dace: distribution-aware counterfactual explanation by mixed-integer linear optimization, in Proceedings of the 29th International Joint Conference on Artificial Intelligence 2855–2862. 10.24963/ijcai.2020/395

[B85] Kantchelian A. Tygar J. D. Joseph A. (2016). Evasion and hardening of tree ensemble classifiers, in Proceedings of the 33rd International Conference on Machine Learning 2387–2396.

[B86] Ke G. Meng Q. Finley T. Wang T. Chen W. Ma W.. (2017). LightGBM: A highly efficient gradient boosting decision tree, in Advances in Neural Information Processing Systems 3149–3157.

[B87] Kiossou H. Nijssen S. Schaus P. Houndji R. (2022). Time constrained dl8.5 using limited discrepancy search, in Machine Learning and Knowledge Discovery in Databases (ECMLPKDD 2022), Part V 443–459. 10.1007/978-3-031-26419-1_2729249083

[B88] Kocev D. Vens C. Struyf J. Dzeroski S. (2013). Tree ensembles for predicting structured output. Patt. Recogn. 46, 817–833. 10.1016/j.patcog.2012.09.023

[B89] Kontschieder P. Fiterau M. Criminisi A. Bulo S. R. (2015). Deep neural decision forests, in Proceedings of the IEEE International Conference on Computer Vision 1467–1475. 10.1109/ICCV.2015.172

[B90] Koschel S. Buschjäger S. Lucchese C. Morik K. (2023). Fast inference of tree ensembles on arm devices. arXiv:2305.08579.

[B91] Kramer S. (1996). Structural regression trees, in Proceedings of the 13th National Conference on Artificial Intelligence 812–819.

[B92] Lee G.-H. Jaakkola T. S. (2020). Oblique decision trees from derivatives of ReLU networks, in International Conference on Learning Representations.

[B93] Levatić J. Ceci M. Kocev D. Džeroski S. (2017). Semi-supervised classification trees. J. Intell. Inform. Syst. 49, 461–486. 10.1007/s10844-017-0457-4

[B94] Li H. Song J. Xue M. Zhang H. Ye J. Cheng L.. (2022). A survey of neural trees. arXiv:2209.03415.

[B95] Li Q. Wu Z. Wen Z. He B. (2020). Privacy-preserving gradient boosting decision trees, in Proceedings of the 34th AAAI Conference on Artificial Intelligence 784–791. 10.1609/aaai.v34i01.542237128389

[B96] Liang C. Zhang Y. Shi P. Hu Z. (2012). Learning very fast decision tree from uncertain data streams with positive and unlabeled samples. Inform. Sci. 213, 50–67. 10.1016/j.ins.2012.05.023

[B97] Lin J. Zhong C. Hu D. Rudin C. Seltzer M. (2020). Generalized and scalable optimal sparse decision trees, in Proceedings of the 37th International Conference on Machine Learning 6150–6160.

[B98] Liu C. Arnon T. Lazarus C. Strong C. Barrett C. Kochenderfer M. J. (2021). Algorithms for verifying deep neural networks. Found. Trends? Optimiz. 4, 244–404. 10.1561/2400000035

[B99] Liu F. T. Ting K. M. Zhou Z.-H. (2008). Isolation forest, in Proceedings of the 8th IEEE International Conference on Data Mining 413–422. 10.1109/ICDM.2008.17

[B100] Lucchese C. Nardini F. M. Orlando S. Perego R. Tonellotto N. Venturini R. (2017). Quickscorer: Efficient traversal of large ensembles of decision trees, in Machine Learning and Knowledge Discovery in Databases (ECMLPKDD 2017), Part III 383–387. 10.1007/978-3-319-71273-4_36

[B101] Lucic A. Oosterhuis H. Haned H. de Rijke M. (2022). Focus: Flexible optimizable counterfactual explanations for tree ensembles, in Proceedings of the 36th AAAI Conference on Artificial Intelligence 5313–5322. 10.1609/aaai.v36i5.20468

[B102] Lundberg S. M. Erion G. Chen H. DeGrave A. Prutkin J. M. Nair B.. (2019). Explainable ai for trees: From local explanations to global understanding. arXiv:1905.04610. 10.1038/s42256-019-0138-9 PMC7326367 32607472

[B103] Madry A. Makelov A. Schmidt L. Tsipras D. Vladu A. (2018). Towards deep learning models resistant to adversarial attacks, in Proceedings of the 6th International Conference on Learning Representations. 34648444

[B104] McTavish H. Zhong C. Achermann R. Karimalis I. Chen J. Rudin C.. (2022). Fast sparse decision tree optimization via reference ensembles, in Proceedings of the 36th AAAI Conference on Artificial Intelligence 9604–9613. 10.1609/aaai.v36i9.21194 36051654 PMC9429834

[B105] Mehta M. Agrawal R. Rissanen J. (1996). Sliq: A fast scalable classifier for data mining, in Advances in Database Technology-EDBT'96: 5th International Conference on Extending Database Technology 18–32. 10.1007/BFb0014141

[B106] Meng Q. Ke G. Wang T. Chen W. Ye Q. Ma Z.-M.. (2016a). A communication-efficient parallel algorithm for decision tree. Adv. Neural Infor. Proc. Syst. 29, 1271–1279.

[B107] Meng X. Bradley J. Yavuz B. Sparks E. Venkataraman S. Liu D.. (2016b). MLlib: *Machine Learning* in Apache Spark. J. Mach. Learn. Res. 17, 1235–1241.

[B108] Milani S. Zhang Z. Topin N. Shi Z. R. Kamhoua C. Papalexakis E. E.. (2022). Maviper: Learning decision tree policies for interpretable multi-agent reinforcement learning, in Machine Learning and Knowledge Discovery in Databases (ECMLPKDD 2022), Part IV 251–266. 10.1007/978-3-031-26412-2_16

[B109] Mitchell R. Frank E. (2017). Accelerating the XGBoost algorithm using GPU computing. PeerJ Comput. Sci. 3, e127. 10.7717/peerj-cs.127

[B110] Murthy S. K. (1998). Automatic construction of decision trees from data: A multi-disciplinary survey. Data Mining Knowl. Disc. 2, 345–389. 10.1023/A:1009744630224

[B111] Murthy S. K. Kasif S. Salzberg S. (1994). A system for induction of oblique decision trees. J. Artif. Intell. Res. 2, 1–32. 10.1613/jair.63

[B112] Nanfack G. Temple P. Frénay B. (2021). Constraint enforcement on decision trees: A survey. ACM Comput. Surv. 54, 1–36. 10.1145/3506734

[B113] Narodytska N. Ignatiev A. Pereira F. Marques-Silva J. (2018). Learning optimal decision trees with SAT, in Proceedings of the 27th International Joint Conference on Artificial Intelligence 1362–1368. 10.24963/ijcai.2018/18935578278

[B114] Nauta M. van Bree R. Seifert C. (2021). Neural prototype trees for interpretable fine-grained image recognition, in Proceedings of the IEEE/CVF Conference on Computer Vision and Pattern Recognition 14933–14943. 10.1109/CVPR46437.2021.01469

[B115] Neville J. Jensen D. D. (2007). Relational dependency networks. J. Mach. Lear. Res. 8, 653–692.

[B116] Nijssen S. Fromont É. (2007). Mining optimal decision trees from itemset lattices, in Proceedings of the 13th ACM SIGKDD International Conference on Knowledge Discovery and Data Mining 530–539. 10.1145/1281192.1281250

[B117] Norouzi M. Collins M. D. Johnson M. Fleet D. J. Kohli P. (2015). Efficient non-greedy optimization of decision trees, in Proceedings of the 28th International Conference on Neural Information Processing Systems 1729–1737.

[B118] Núñez M. (1991). The use of background knowledge in decision tree induction. Mach. Lear. 6, 231–250. 10.1007/BF00114778

[B119] Okajima Y. Sadamasa K. (2019). Deep neural networks constrained by decision rules, in Proceedings of the 33th AAAI Conference on Artificial Intelligence 2496–2505. 10.1609/aaai.v33i01.3301249636624117

[B120] Olaru C. Wehenkel L. (2003). A complete fuzzy decision tree technique. Fuzzy Sets Syst. 138, 221–254. 10.1016/S0165-0114(03)00089-7

[B121] Parmentier A. Vidal T. (2021). Optimal counterfactual explanations in tree ensembles, in Proceedings of the 38th International Conference on Machine Learning 8422–8431.

[B122] Potharst R. Feelders A. J. (2002). Classification trees for problems with monotonicity constraints. SIGKDD Explor. 4, 1–10. 10.1145/568574.568577

[B123] Provost F. J. Domingos P. M. (2003). Tree induction for probability-based ranking. Mach. Lear. 52, 199–215. 10.1023/A:1024099825458

[B124] Quinlan J. R. (1986). Induction of decision trees. Mach. Lear. 1, 81–106. 10.1007/BF00116251

[B125] Quinlan J. R. (1992). Learning with continuous classes, in Proceedings of the 5th Australian Joint Conference on Artificial Intelligence 343–348.

[B126] Ram P. Gray A. G. (2011). Density estimation trees, in Proceedings of the 17th ACM SIGKDD International Conference on Knowledge Discovery and Data Mining, page 627–635. 10.1145/2020408.2020507

[B127] Ranzato F. Zanella M. (2020). Abstract interpretation of decision tree ensemble classifiers, in Proceedings of the AAAI Conference on Artificial Intelligence 5478–5486. 10.1609/aaai.v34i04.5998

[B128] Ranzato F. Zanella M. (2021). Genetic adversarial training of decision trees, in Proceedings of the 2021 Genetic and Evolutionary Computation Conference 358–367. 10.1145/3449639.3459286

[B129] Ribeiro M. T. Singh S. Guestrin C. (2016a). Model-agnostic interpretability of machine learning, in ICML Workshop on Human Interpretability in Machine Learning, WHI '16 (Stockholm, Sweden).

[B130] Ribeiro M. T. Singh S. Guestrin C. (2016b). ‘Why should I trust you?': Explaining the predictions of any classifier, in Proceedings of the 22nd ACM SIGKDD International Conference on Knowledge Discovery and Data Mining 1135–1144. 10.1145/2939672.2939778

[B131] Rokach L. (2016). Decision forest: Twenty years of research. Inf. Fusion 27, 111–125. 10.1016/j.inffus.2015.06.005

[B132] Rota Bulo S. Kontschieder P. (2014). Neural decision forests for semantic image labelling, in Proceedings of the 2014 IEEE Conference on Computer Vision and Pattern Recognition 81–88. 10.1109/CVPR.2014.18

[B133] Sagi O. Rokach L. (2018). Ensemble learning: A survey. WIREs Data Min. Knowl. Disc. 8, e1249. 10.1002/widm.1249

[B134] Sato N. Kuruma H. Nakagawa Y. Ogawa H. (2020). Formal verification of a decision-tree ensemble model and detection of its violation ranges. IEICE Trans. Inf. Syst. E103, 363–378. 10.1587/transinf.2019EDP7120

[B135] Shafer J. C. Agrawal R. Mehta M. (1996). Sprint: A scalable parallel classifier for data mining, in Proceedings of the 22th International Conference on Very Large Data Bases 544–555.

[B136] Sharp T. (2008). Implementing decision trees and forests on a gpu, in Computer Vision-ECCV 2008, Part IV, Lecture Notes in Computer Science (Springer) 595–608. 10.1007/978-3-540-88693-8_44

[B137] Shi Y. Ke G. Chen Z. Zheng S. Liu T.-Y. (2022). Quantized training of gradient boosting decision trees, in Advances in Neural Information Processing Systems 35. PMC10544768 37786624

[B138] Strobl C. Boulesteix A.-L. Zeileis A. Hothorn T. (2007). Bias in random forest variable importance measures: Illustrations, sources and a solution. BMC Bioinform. 8, 25. 10.1186/1471-2105-8-25 17254353 PMC1796903

[B139] Struyf J. Džeroski S. (2006). Constraint based induction of multi-objective regression trees, in Proceedings of the 4th International Conference on Knowledge Discovery in Inductive Databases, KDID'05 222–233. 10.1007/11733492_13

[B140] Szegedy C. Zaremba W. Sutskever I. Bruna J. Erhan D. Goodfellow I.. (2013). Intriguing properties of neural networks. arXiv:1312.6199.

[B141] Todorovski L. Blockeel H. Džeroski S. (2002). Ranking with predictive clustering trees, in Proceedings of the 13th European Conference on Machine Learning 444–455. 10.1007/3-540-36755-1_37

[B142] Tolomei G. Silvestri F. Haines A. Lalmas M. (2017). Interpretable predictions of tree-based ensembles via actionable feature tweaking, in Proceedings of the 23rd ACM SIGKDD International Conference on Knowledge Discovery and Data Mining 465–474. 10.1145/3097983.3098039

[B143] Törnblom J. Nadjm-Tehrani S. (2020). Formal verification of input-output mappings of tree ensembles. Sci. Comput. Program. 194, 102450. 10.1016/j.scico.2020.102450

[B144] Tyree S. Weinberger K. Q. Agrawal K. Paykin J. (2011). Parallel boosted regression trees for web search ranking, in Proceedings of the 20th international conference on World Wide Web 387–396. 10.1145/1963405.1963461

[B145] Utgoff P. E. (1989). Incremental induction of decision trees. Mach. Lear. 4, 161–186. 10.1023/A:1022699900025

[B146] van der Linden J. G. Weerdt M. Demirović E. (2022). Fair and optimal decision trees: A dynamic programming approach, in Advances in Neural Information Processing Systems 35.

[B147] Van Essen B. Macaraeg C. Gokhale M. Prenger R. (2012). Accelerating a random forest classifier: Multi-Core, GP-GPU, or FPGA? in 2012 IEEE 20th International Symposium on Field-Programmable Custom Computing Machines 232–239. 10.1109/FCCM.2012.47

[B148] Van Wolputte E. Blockeel H. (2020). Missing value imputation with MERCS: A faster alternative to missforest, in Proceedings of the 23rd International Conference, on Discovery Science 502–516. 10.1007/978-3-030-61527-7_33

[B149] Van Wolputte E. Korneva E. Blockeel H. (2018). MERCS: multi-directional ensembles of regression and classification trees, in Proceedings of the 32nd AAAI Conference on Artificial Intelligence 4276–4283. 10.1609/aaai.v32i1.11735

[B150] Vens C. Struyf J. Schietgat L. Dzeroski S. Blockeel H. (2008). Decision trees for hierarchical multi-label classification. Mach. Lear. 73, 185–214. 10.1007/s10994-008-5077-325865524

[B151] Verhaeghe H. Nijssen S. Pesant G. Quimper C. Schaus P. (2020). Learning optimal decision trees using constraint programming. Constr. Int. J. 25, 226–250. 10.1007/s10601-020-09312-3

[B152] Verwer S. Zhang Y. (2019). Learning optimal classification trees using a binary linear program formulation, in Proceedings of the 33rd AAAI Conference on Artificial Intelligence 1625–1632. 10.1609/aaai.v33i01.33011624

[B153] Vos D. Verwer S. (2021). Efficient training of robust decision trees against adversarial examples, in Proceedings of the 38th International Conference on Machine Learning 10586–10595.

[B154] Vos D. Verwer S. (2022). Robust optimal classification trees against adversarial examples, in Proceedings of the 36th AAAI Conference on Artificial Intelligence 8520–8528. 10.1609/aaai.v36i8.20829

[B155] Vos D. Verwer S. (2023). Adversarially robust decision tree relabeling, in Machine Learning and Knowledge Discovery in Databases (ECMLPKDD 2022), Part III 203–218. 10.1007/978-3-031-26409-2_13

[B156] Wang Y. Zhang H. Chen H. Boning D. Hsieh C.-J. (2020). On Lp-norm robustness of ensemble decision stumps and trees, in Proceedings of the 37th International Conference on Machine Learning 10104–10114.

[B157] Wei D. Nair R. Dhurandhar A. Varshney K. R. Daly E. M. Singh M. (2022). On the safety of interpretable machine learning: A maximum deviation approach, in Advances in Neural Information Processing Systems 35.

[B158] Wen Z. He B. Kotagiri R. Lu S. Shi J. (2018). Efficient gradient boosted decision tree training on GPUs, in 2018 IEEE International Parallel and Distributed Processing Symposium (IPDPS) 234–243. 10.1109/IPDPS.2018.00033

[B159] Wu G. Li H. Hu X. Bi Y. Zhang J. Wu X. (2009). Mrec4.5: C4.5 ensemble classification with mapreduce, in 2009 Fourth ChinaGrid Annual Conference 249–255.

[B160] Wu M. Hughes M. Parbhoo S. Zazzi M. Roth V. Doshi-Velez F. (2018). Beyond sparsity: Tree regularization of deep models for interpretability, in Proceedings of the AAAI conference on artificial intelligence 32. 10.1609/aaai.v32i1.11501

[B161] Xie L. Liu J. Lu S. Chang T.-H. Shi Q. (2022). An efficient learning framework for federated XGBoost using secret sharing and distributed optimization. ACM Trans. Intell. Syst. Technol. 13, 1–28. 10.1145/3523061

[B162] Xu R. Baracaldo N. Joshi J. (2021). Privacy-preserving machine learning: Methods, challenges and directions. arXiv:2108.04417.

[B163] Xu Z. Zhu G. Yuan C. Huang Y. (2022). One-stage tree: end-to-end tree builder and pruner. Mach. Lear. 111, 1959–1985. 10.1007/s10994-021-06094-4

[B164] Yang Y.-Y. Rashtchian C. Zhang H. Salakhutdinov R. R. Chaudhuri K. (2020). A closer look at accuracy vs. robustness, in Advances in Neural Information Processing Systems 8588–8601.

[B165] Ye T. Zhou H. Zou W. Y. Gao B. Zhang R. (2018). Rapidscorer: Fast tree ensemble evaluation by maximizing compactness in data level parallelization, in Proceedings of the 24th ACM SIGKDD International Conference on Knowledge Discovery &Data Mining 941–950.

[B166] Yu P. L. H. Wan W. M. Lee P. H. (2011). Preference Learning, chapter Decision Tree Modeling for Ranking Data. (Berlin: Springer) 83–106. 10.1007/978-3-642-14125-6_5

[B167] Zantedeschi V. Kusner M. Niculae V. (2021). Learning binary decision trees by argmin differentiation, in Proceedings of the 38th International Conference on Machine Learning 12298–12309.

[B168] Ženko B. Todorovski L. Džeroski S. (2001). A comparison of stacking with meta decision trees to bagging, boosting, and stacking with other methods, in Proceedings of the 2001 IEEE International Conference on Data Mining 669–670.

[B169] Zhang C. Zhang H. Hsieh C.-J. (2020a). An efficient adversarial attack for tree ensembles, in Advances in Neural Information Processing Systems 16165–16176.

[B170] Zhang F. Wang Y. Liu S. Wang H. (2020b). Decision-based evasion attacks on tree ensemble classifiers. World Wide Web 23, 2957–2977. 10.1007/s11280-020-00813-y

[B171] Zhang H. Si S. Hsieh C.-J. (2017). Gpu-acceleration for large-scale tree boosting. arXiv:1706.08359.

[B172] Zhang W. Ntoutsi E. (2019). FAHT: an adaptive fairness-aware decision tree classifier, in Proceedings of the 28th International Joint Conference on Artificial Intelligence 1480–1486. 10.24963/ijcai.2019/205

[B173] Zhou Y. McArdle J. J. (2015). Rationale and applications of survival tree and survival ensemble methods. Psychometrika 3, 811–833. 10.1007/s11336-014-9413-1 25228495 PMC4409541

[B174] Zhou Z.-H. (2012). Ensemble Methods: Foundations and Algorithms. 1st edition. London: Chapman &Hall/CRC. 10.1201/b12207

